# Machine learning‐based SERS serum detection platform for high‐sensitive and high‐throughput diagnosis of colorectal precancerous lesions

**DOI:** 10.1002/btm2.70019

**Published:** 2025-03-28

**Authors:** Qunshan Zhu, Gaoyang Chen, Lei Fu, Dawei Cao, Zhenguang Wang, Yan Yang, Wei Wei

**Affiliations:** ^1^ Department of Gastrointestinal Surgery Jiangdu People's Hospital Affiliated to Yangzhou University Yangzhou China; ^2^ Department of Oncology The Affiliated Taizhou Second People's Hospital of Yangzhou University Taizhou China; ^3^ Department of Pathology Jiangdu People's Hospital Affiliated to Yangzhou University Yangzhou China; ^4^ School of Information Engineering Yangzhou Polytechnic Institute Yangzhou China

**Keywords:** colorectal cancer, colorectal precancerous lesions, machine learning, principal component analysis, surface‐enhanced Raman scattering

## Abstract

Colorectal precancerous lesions (CRP) are early signs of cancer development, and early detection helps prevent progression to colorectal cancer (CRC), reducing incidence and mortality rates. This study developed a serum detection platform integrating surface‐enhanced Raman scattering (SERS) with machine learning (ML) for early detection of CRP. Specifically, a microarray chip with Au/SnO_2_ nanorope arrays (Au/SnO_2_ NRAs) substrate was designed for SERS spectral measurement of serum. The Principal Component Analysis (PCA)‐Optimal Class Discrimination and Compactness Optimization (OCDCO) model was proposed to identify CRP spectra. The results demonstrated that the microarray chip exhibited superior portability, SERS activity, stability, and uniformity. Through PCA‐OCDCO, the serum samples from healthy controls, CRP patients, and CRC patients were effectively classified, and several key spectral features for distinguishing different groups were identified. The established PCA‐OCDCO model achieved outstanding performance, with an accuracy of 97%, a sensitivity of 95%, a specificity of 97%, and an AUC of 0.96. This study suggests that the platform, integrating SERS with the PCA‐OCDCO model, holds potential for the early detection of CRP, providing an approach for CRP prevention and clinical diagnostics.

**Translational Impact Statement**

This study introduced a novel serum detection platform integrating SERS and ML for the early detection of CRP. The developed microarray chip with Au/SnO_2_ NRAs substrate exhibited good portability, high throughput, SERS activity, stability, and uniformity. The PCA‐OCDCO model achieved high accuracy, sensitivity, specificity, and AUC in distinguishing serum samples from healthy controls, CRP patients, and CRC patients. By identifying key spectral features for differentiation, this platform holds great potential for early CRP detection and advancing clinical diagnostics.

## 1 INTRODUCTION

Colorectal cancer (CRC) is one of the most prevalent and deadly cancers worldwide, with over 1.8 million new cases diagnosed annually and nearly 900,000 deaths each year, according to the World Health Organization (WHO).^1^, ^2^, ^3^ CRC typically originates from colorectal precancerous lesions (CRP), such as adenomas and polyps, which can gradually develop into malignant tumors.^4^ Early detection of CRP is crucial for preventing CRC and reducing its global health impact. Currently, the diagnostic methods for CRP mainly include colonoscopy and fecal occult blood tests, but each has its limitations.^5^ Colonoscopy, the gold standard, allows direct visualization of polyps or adenomas but is invasive, uncomfortable, and expensive, reducing patient compliance.^6^ Fecal occult blood tests are non‐invasive and affordable but lack sensitivity and specificity, leading to false results and missing.^7^ In summary, the current diagnostic methods are inadequate for early detection of CRP. A rapid, label‐free, high‐accuracy, and high‐throughput technology is urgently needed. Serum samples are commonly used in clinical diagnostics as they contain proteins, carbohydrates, lipids, enzymes, nucleic acids, and other biomolecules that offer key insights into disease progression.^8^, ^9^ In CRC research, analyzing the changes in these serum components can reveal biomarkers and pathological mechanisms of disease progression, providing a valuable tool for CRP diagnosis and intervention.

Surface‐enhanced Raman scattering (SERS) has a wide range of applications across various fields.^10^, ^11^ In the medical field, SERS has shown remarkable potential.^12^ Li et al. used SERS for the early detection of breast cancer by identifying specific biomarkers in blood samples.^13^ Chen et al. utilized SERS to detect pathogenic bacteria in wound infections, significantly reducing diagnosis time and improving treatment accuracy.^14^ SERS shows great potential in improving diagnostics. Raman scattering (RS) is a frequency shift phenomenon that occurs when light interacts with molecules, revealing the molecular structure of substances.^15^, ^16^ It offers high resolution, non‐destructive analysis, making it widely applicable for analyzing biological samples such as blood and urine. However, Raman signals are typically weak and can be easily interfered with by fluorescence and background noise, especially in complex samples.^17^ SERS is based on Raman, significantly enhancing signals through local surface plasmon resonance in metal nanostructures like gold or silver.^18^ The advantages of SERS include greatly improved sensitivity, enhanced signal‐to‐noise ratio, and the ability to analyze very low concentrations of analytes.^19^ The high sensitivity and signal enhancement capability of SERS depend largely on the nanoscale structural characteristics of the sensor surface. The design of the surface morphology directly determines the enhancement effect. The morphology of Au/SnO_2_ nanorope arrays (Au/SnO_2_ NRAs) shows densely packed nanorods and uniformly distributed nanopores.^10^, ^11^ This structure significantly enhances the SERS effect, as the gaps between the nanorods and nanopores create numerous “hotspots,” which greatly amplify the Raman signal through local surface plasmon resonance.^12^ The prepared Au/SnO_2_ NRAs substrate is an excellent SERS material.

Serum is rich in biomolecules, providing critical disease information, but air exposure can cause degrading samples and affect accuracy.^13^, ^14^ Microarray chips enhance SERS signals and prevent environmental interference with their integrated substrate and enclosed design. Microarray chips integrate the SERS substrate into the chip, significantly enhancing the SERS signal, while their enclosed design effectively prevents environmental interference.^20^ This chip can achieve highly sensitive and reproducible SERS signal detection, ensuring efficient performance in complex serum samples.^21^ The advancement of Artificial Intelligence (AI) enables precise analysis of SERS data, using machine learning (ML) to identify disease and biological characteristics.^22^, ^23^, ^24^ SERS combined with ML has demonstrated significant advancements in disease diagnostics.^25^ One study utilized Principal Component Analysis (PCA) with SERS to detect precancerous gastric lesions, improving early diagnosis accuracy.^26^ Another study applied the K‐Nearest Neighbor (KNN) algorithm to SERS data for the precise identification of bacterial infections, enhancing diagnostic precision.^27^ The KNN algorithm is based on the distance to labeled data, making it susceptible to noise.^28^ The Support Vector Machines (SVM) constructs an optimal hyperplane but has limitations with nonlinear data.^29^ The Optimal Class Discrimination and Compactness Optimization (OCDCO) algorithm maximizes the separation of different classes while optimizing the compact clustering of data points within the same class, thereby enhancing classification accuracy and robustness.

**FIGURE 1:**
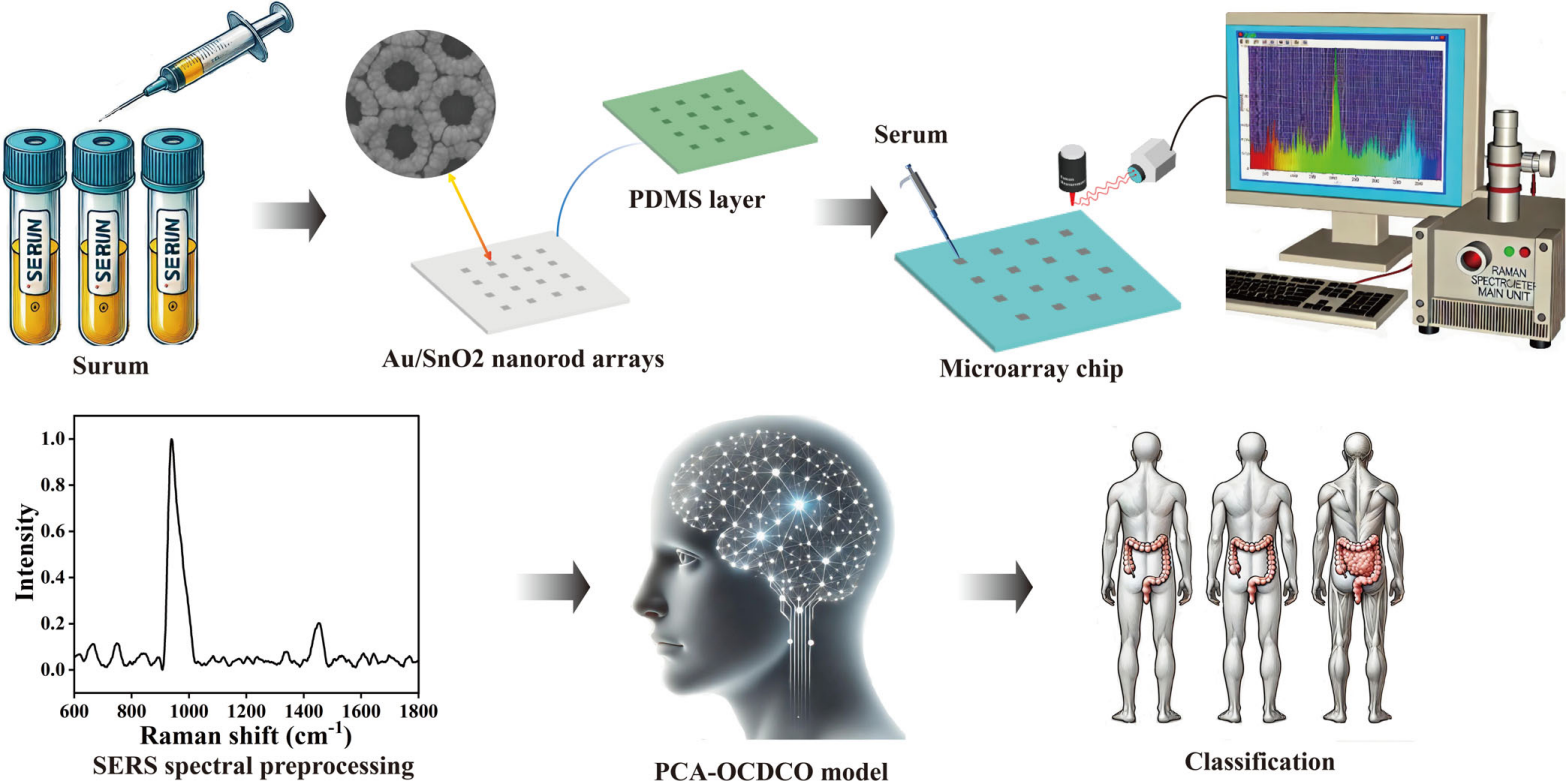
A detection platform combining ML and SERS technology for CRP.

In this study, we designed a detection platform combining ML and SERS technology for CRP, as shown in Figure 1. First, serum samples were collected from subjects, including healthy control (HC), CRP, and CRC. Second, the microarray chip with an Au/SnO_2_ NRAs substrate was designed and used for serum detection. Third, the PCA‐OCDCO model was proposed to construct a SERS spectral identification model. The results showed that its prediction accuracy, sensitivity, specificity, and Area Under the Curve(AUC) were excellent, achieving rapid and efficient CRP detection.

## 2 MATERIALS AND METHODS

### 2.1 Materials

5,5′‐Dithiobis (2‐nitrobenzoic acid) (DTNB) and polydimethylsiloxane (PDMS) were sourced from Shanghai Aladdin Biochemical Technology Co., Ltd.; Tin (IV) chloride (SnCl4) and anhydrous ethanol were provided by China National Pharmaceutical Group Chemical Reagent Co., Ltd.; and the polystyrene microsphere (PS) suspension (5 wt%) was procured from Zhongke LanYing (Beijing) Technology Co., Ltd. All glassware used in the experiments was cleaned by immersion in aqua regia and thoroughly rinsed with deionized water, which had a resistivity of 18.2 MΩ throughout the experiments.

### 2.2 Instrumentation

Magnetic stirring was carried out using a magnetic stirrer from Tianjin Top Instrument Co., Ltd. The high‐temperature annealing was performed in a box furnace from Changsha Keli Technology Co., Ltd. Electroplating was conducted with a constant current electroplating instrument from Nanjing Kehua Instrument Co., Ltd. Centrifugation was done using a 5810R centrifuge from Eppendorf (Germany). Field emission scanning electron microscope (SEM) imaging was performed using a JSM‐7800F field emission microscope from JEOL Ltd. (Japan). Raman spectroscopy was conducted with a Thermo Scientific DXR Raman spectrometer from Thermo Fisher Scientific Inc. (USA), and UV–vis spectra were recorded with a Lambda 950 spectrophotometer from PerkinElmer, Inc. (USA).

### 2.3 Synthesis of Au/SnO_2_ NRAs

First, the SnCl_4_ solution with a concentration of 0.05 M was configured to ensure that the PS microspheres (500 nm diameter) were fully infiltrated. The gas–liquid interface self‐assembly method was used to obtain PS sphere monolayer templates. The obtained PS sphere templates were annealed at 400°C for 2 h to obtain SnO_2_ nanobowl arrays (SnO_2_ NBAs). Setting the current to 30 mA, the SnO_2_ NBAs were treated with Au deposition for 8 min, and the Au/SnO_2_ NRAs were successfully prepared.

### 2.4 Fabrication of microarray chips

To achieve high sensitivity and high throughput in serum analysis, we initiated the process by designing and drafting the microwell chip layout using SolidWorks software. Subsequently, a mold was fabricated using soft lithography techniques. Following the etching process, a PDMS and curing agent mixture in a 12:1 ratio was poured into the mold and allowed to cure on a hot plate at 75°C for 2 h. After curing and cooling to room temperature, the PDMS was perforated and subjected to ultrasonic cleaning. Post‐cleaning and drying, both the PDMS and glass slides underwent plasma treatment. The SnO_2_ NRAs substrate was then integrated into the hydrophilic PDMS chip, finalizing the fabrication of the SERS microarray chip. The resulting chip, measuring 27 × 27 mm, comprised 16 SnO_2_ NRAs substrates, each with a diameter of 4 mm. This fabrication method ensured high efficiency and reliability, making the chip well suited for high‐throughput serum analysis.

### 2.5 SERS measurement and characterization

2 μL serum was accurately dispensed into each microwell of the microarray chip. The SERS spectra were obtained using a Thermo Scientific DXR Raman microscope spectrometer equipped with a 785 nm HeNe laser and a 50× objective lens. The laser power was adjusted to 5 mW, with a spot size of 2 μm and an acquisition time of 10 s per measurement. Spectral data were recorded over a range of 600 to 1800 cm^−1^. To prevent interference from cosmic rays, negative peaks, or sharp positive peaks, all experiments were conducted in a dark room. For each sample, spectra were measured from five distinct locations within the SERS‐active region, and the data were averaged. This averaging process helped to mitigate the effects of local inhomogeneities on the substrate, providing more consistent and representative results.

### 2.6 Data preprocessing and multivariate analysis

After collecting the original SERS spectra, they were preprocessed using Origin 2019. First, the Savitzky–Golay filter (window length of 11, polynomial order of 3) was applied to smooth the spectra and reduce noise. Then, the Asymmetric Least Squares Smoothing algorithm (smoothing parameter 1 × e^6^, asymmetry parameter 0.01) was used for baseline correction to remove background fluorescence. Finally, min‐max normalization was performed to scale the spectral intensities between 0 and 1. Afterward, the spectra were averaged to analyze spectral characteristics. PCA was then performed on the preprocessed spectra of HC, CRP, and CRC samples. Using Origin 2019, the PCA score plot, scree plot, loading plot, and scatter plot were generated. Subsequently, the first 12 PCs were used as inputs for OCDCO. Matlab 2020 was employed to conduct 5‐fold cross‐validation to assess the accuracy, sensitivity, specificity, and AUC of the PCA‐OCDCO model.

## 3 RESULTS AND DISCUSSION

### 3.1 Characterization of microarray chip

**FIGURE 2:**
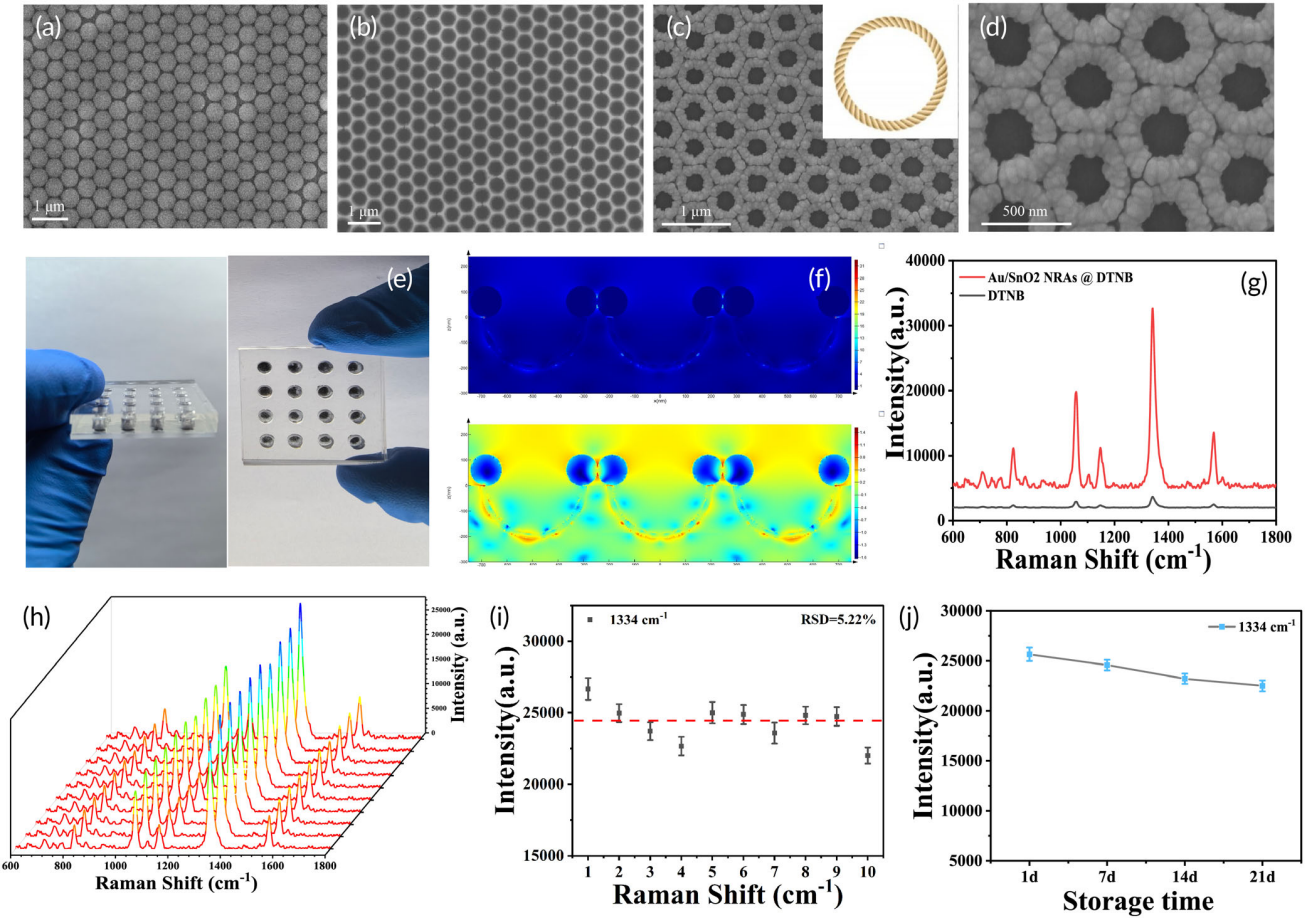
(a) SEM image of the PS microsphere template. (b) SEM image of the intermediate product SnO_2_ NBAs. (c, d) SEM images of Au/SnO_2_ NRAs at different magnifications. (e) Physical image of microarray chip. (f) FDTD simulation image of Au/SnO_2_ NRAs at different magnifications. (g) Raman spectra of DTNB‐labeled Au/SnO_2_ NRAs. (h) All SERS spectra of 10 random points on the surface and (i) intensity histogram at 1334 cm^−1^. (j) Storage stability assessment.

Figure 2a shows the SEM image of a monolayer template of PS submerged by a low concentration of SnCl_4_ solution, and the PS microspheres were neatly aligned and highly ordered. Figure 2b shows SEM images of SnO_2_ NBAs, an intermediate product of the preparation process, showing the regular bowl‐like structure. SEM images of Au/SnO_2_ NRAs at low magnification (Figure 2c) and high magnification (Figure 2d) show that the Au/SnO_2_ NRAs are regular, ordered, and in close contact, forming the ring‐like structure with the rough surface that resembles a rope. Au/SnO_2_ NRAs show densely packed nanorods and uniformly distributed nanopores. These hotspots enhanced the SERS effect through localized surface plasmon resonance (LSPR), generating intense electromagnetic fields and significantly amplifying Raman signals. The prepared microarray chip measured 27 × 27 mm and contained 16 Au/SnO_2_ NRA substrates with a diameter of 4 mm each (Figure 2e). The chip ensured the efficiency and reliability of SERS detection, making it suitable for high‐throughput serum analysis. The electromagnetic field distribution of Au/SnO_2_ NRAs in the vertical direction was simulated using the finite‐difference time‐domain (FDTD) method (Figure 2f), and the bowl structure as the intermediates provided a large number of “hot spots” for the Au/SnO_2_ NRAs obtained by subsequent Au deposition. The Au/SnO_2_ NRAs were labeled using DTNB, and the SERS enhancement effect of Au/SnO_2_ NRAs was excellent with an EF value of 1.67 × 10^8^ according to the formula EF = (I_SERS_/C_SERS_)/(I_Raman_/C_Raman_) (Figure 2g). Ten random points on the Au/SnO_2_ NRAs were taken for SERS measurements, and all the spectra were obtained (Figure 2h), and the Raman characteristic peak at 1334 cm^−1^ was selected to plot the histogram of SERS intensities of the random 10 points (Figure 2i), and the relative standard deviation (RSD) obtained from the calculations was 5.22%, with a good homogeneity of the Au/SnO_2_ NRAs. When the DTNB‐labeled Au/SnO_2_ NRAs were placed at room temperature for several days (1, 7, 14, and 21 days), the intensity of the characteristic peak at 1334 cm^−1^ decreased by only 12.32% (Figure 2j), indicating that the SERS enhancement performance could still be maintained well after prolonged storage and the storage stability was excellent.

### 3.2 Characterization of HC, CRP, and CRC

**FIGURE 3:**
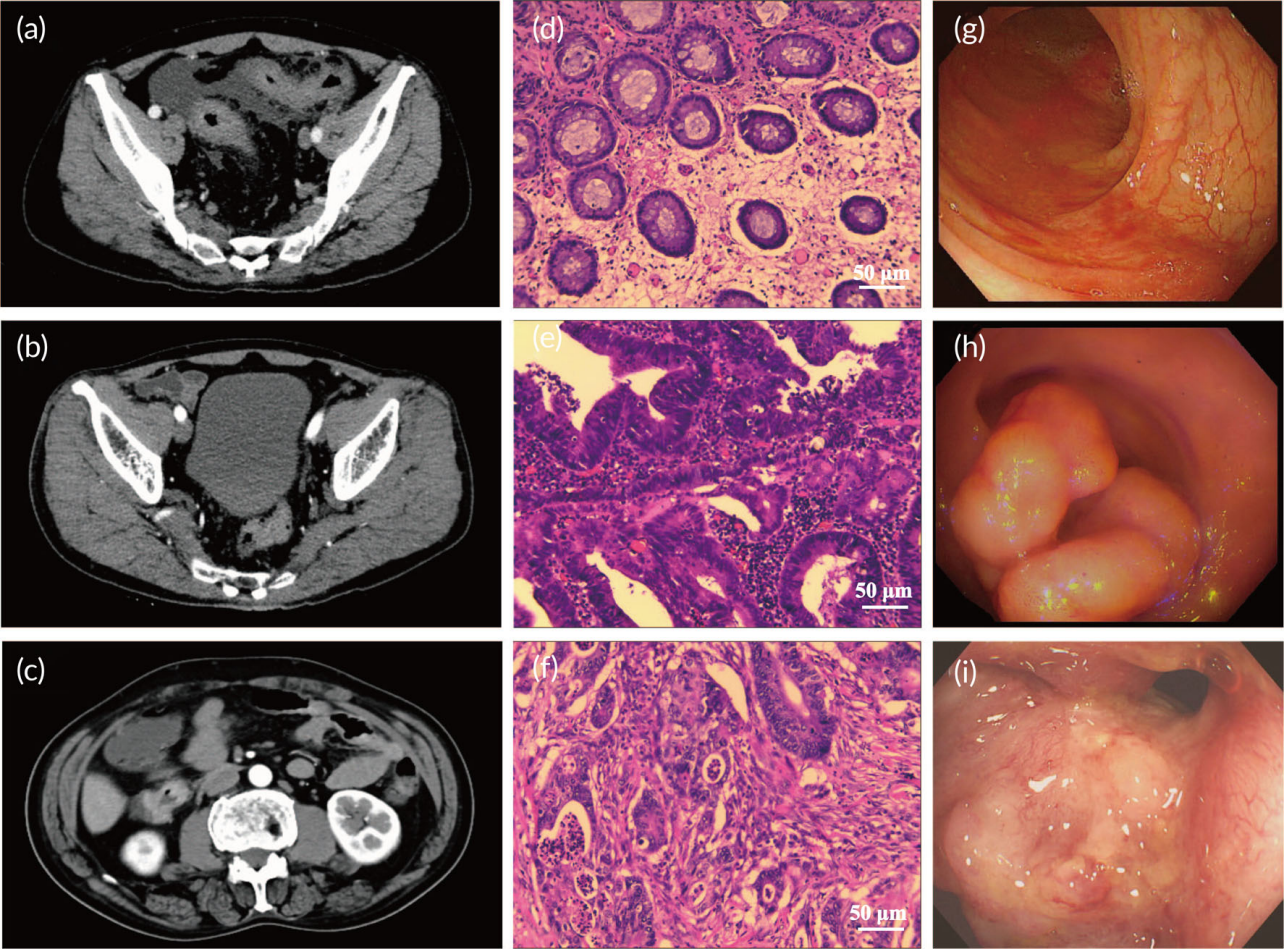
The CT scan of (a) HC, (b) CRP, and (c) CRC. The HE staining of (d) HC, (e) CRP, and (f) CRC. The colonoscopy image of (g) HC, (h) CRP, and (i) CRC.

Serum samples were obtained from subjects who consented in accordance with the ethical standards of the Council for International Organizations of Medical Sciences. The study procedures conformed to the Declaration of Helsinki and received ethical approval from the Ethics Committee of Jiangdu People's Hospital Affiliated to Yangzhou University. Serum samples were collected from 30 HC subjects, 30 CRP patients, and 40 CRC patients. All subjects underwent Computed Tomography (CT), Histological Section (HE) staining, and colonoscopy (Figure 3). In HC subjects, the CT scan (Figure 3a) showed a normal abdominal structure with a thin, even colonic wall. The HE staining (Figure 3d) displayed normal colonic mucosa with well‐organized glands and no dysplasia, and colonoscopy (Figure 3g) revealed smooth mucosa with a uniform reddish color and no visible lesions. In CRP patients, the CT scan (Figure 3b) revealed mild colonic wall thickening, suggesting early‐stage changes. HE staining (Figure 3e) showed mild to moderate dysplasia with elongated, hyperchromatic nuclei, while colonoscopy (Figure 3h) highlighted areas of redness, shallow ulcers, and irregular mucosa. In CRC patients, the CT scan (Figure 3c) revealed pronounced colonic wall thickening and possible tumor presence. HE staining (Figure 3f) displayed severe dysplasia with loss of glandular structure and invasive growth, while colonoscopy (Figure 3i) showed large, irregular ulcers and significant mucosal destruction.

### 3.3 SERS spectral analysis of serum samples from HC, CRP, and CRC

**FIGURE 4:**
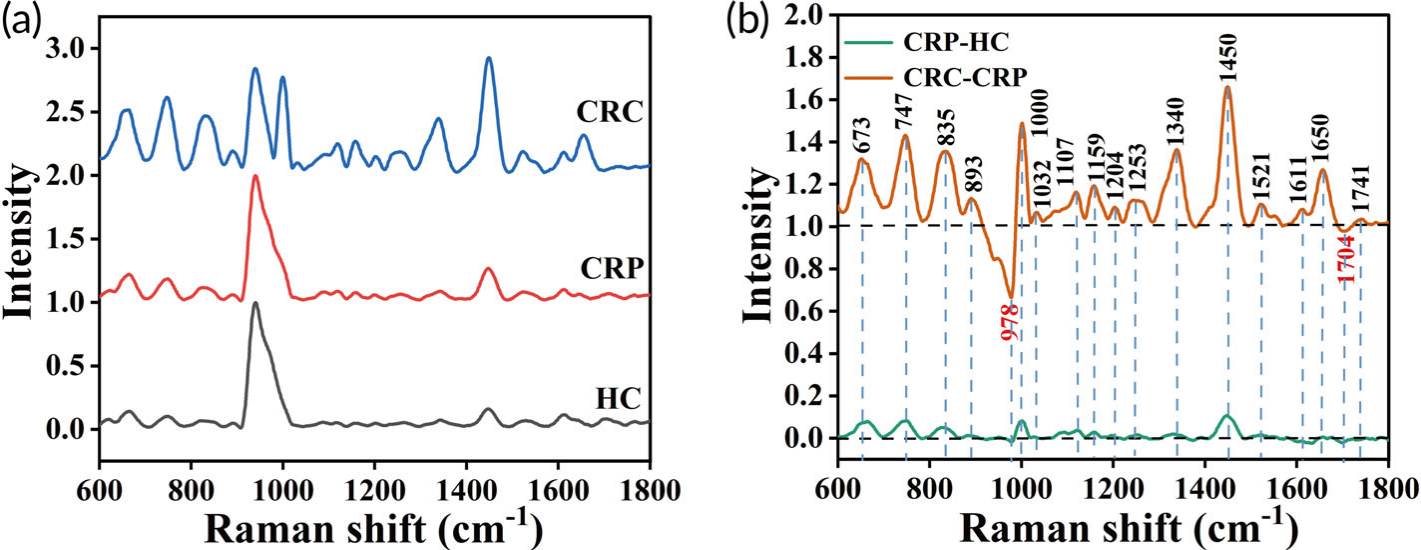
(a) Average SERS spectra of serum samples from HC, CRP, and CRC. (b) Differential SERS Spectra of serum samples (CRC‐CRP and CRP‐HC).

The spectra of 30 HC, 30 CRP, and 40 CRC samples were averaged.^30^ Figure 4a shows the average SERS spectra of HC, CRP, and CRC. From the figure, it could be observed that as the disease progressed, the overall intensity of the spectra gradually increased, and the characteristic peaks became more pronounced. A slight horizontal shift in the characteristic peaks was also observed, which could be attributed to sample variability or minor differences in experimental conditions. However, this shift does not affect the overall correlation between spectral features and disease progression. The characteristic peaks can effectively reflect the development and pathological changes of CRC, providing a clear correlation between spectral features and disease progression. Figure 4b shows the differential SERS spectra between HC, CRP, and CRC. The differential SERS spectra were obtained by comparing the average SERS spectra, reflecting the good reproducibility of the method. From the figure, it could be observed that these characteristic peaks, including 673, 747, 835, 893,^31^ 978, 1000, 1032, 1107, 1159, 1204, 1253,^32^ 1340, 1450, 1521,^33^ 1650, 1704,^34^ and 1741 cm^−1^, showed significant differences between HC, CRP, and CRC, reflecting specific molecular structural and compositional changes of serum. The detailed assignments of characteristic peaks are shown in Table S1. Amino acid‐related peaks included 673 cm^−1^ (ring breathing modes in the DNA bases guanine (G)),^35^ 747 cm^−1^ (ring breathing mode of DNA/RNA bases thymine (T)),^35^ 835 cm^−1^ (asymmetric O‐P‐O stretching, tyrosine),^35^ 1000 cm^−1^ (phenylalanine, bound & free NADH),^36^ and 1204 cm^−1^ (tyrosine, phenylalanine).^37^ The changes in these peaks may be due to DNA damage, alterations in gene expression, and changes in protein metabolism during the progression of cancer. Protein‐related peaks were mainly 978 cm^−1^ (C–C stretching in β‐sheet proteins, =CH bending in lipids),^38^ 1032 cm^−1^ (CH_2_CH_3_ bending modes of collagen & phospholipids, phenylalanine of collagen),^39^, ^40^ 1107 cm^−1^ (phenylalanine),^41^ and 1159 cm^−1^ (C‐C/C‐N stretching).^38^ The enhancement of these peaks indicated changes in protein synthesis and structure, likely due to the increased metabolic demands and rapid growth of cancer cells. The nucleic acid‐related peak was 1340 cm^−1^ (nucleic acid modes),^34^ with its increased intensity reflecting a rise in nucleic acid content, consistent with the high proliferation rate of cancer cells. Lipid‐related peaks included 1741 cm^−1^ (carbonyl feature of lipids),^42^ with changes indicating significant alterations in lipid metabolism, potentially affecting cell membrane integrity and signal transduction. These characteristic peaks provided important molecular evidence for the diagnosis and pathological study of CRP.

### 3.4 Multivariate analysis

**FIGURE 5:**
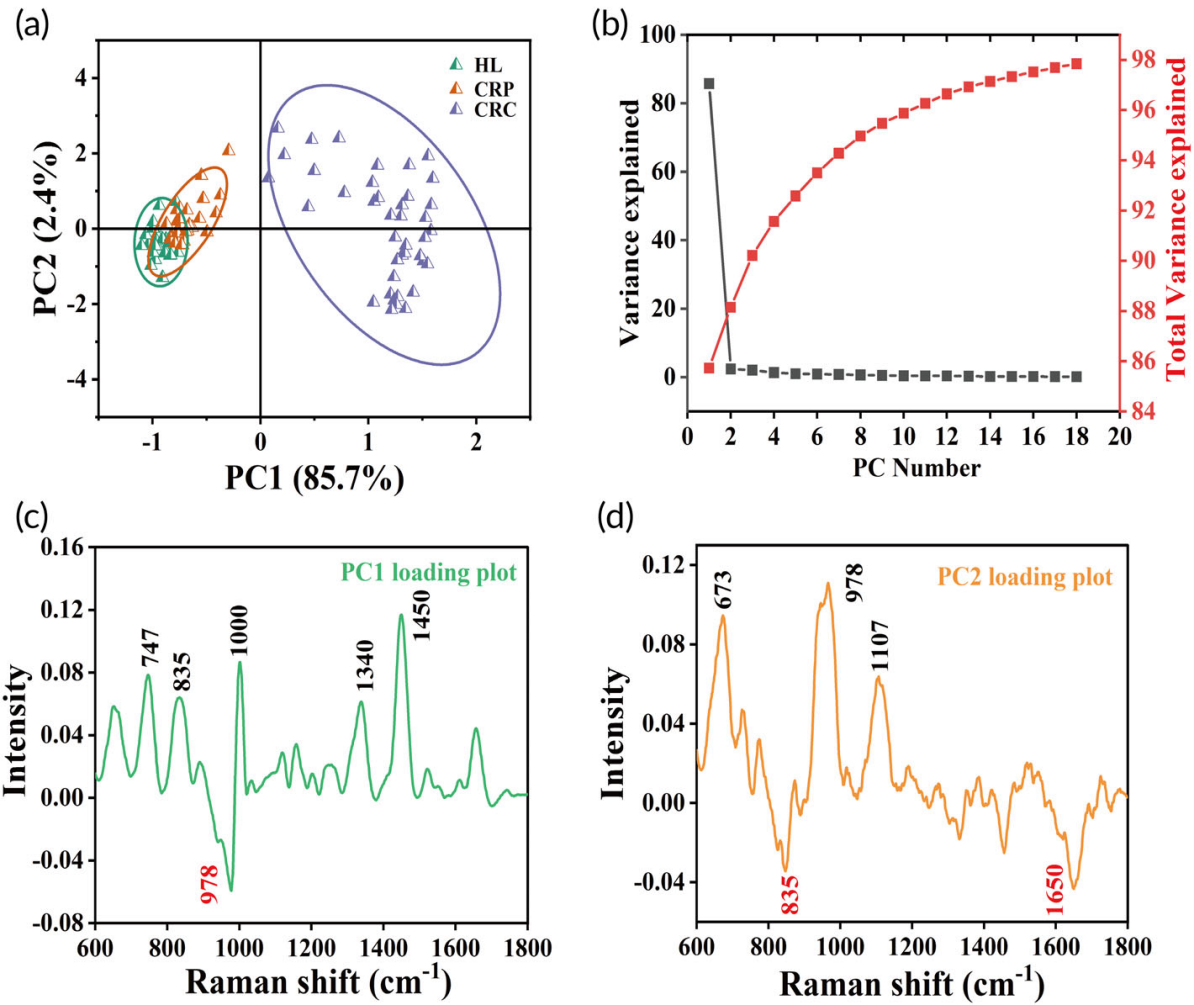
(a) The PCA score plot for HC, CRP, and CRC classes. (b) The plot of variance explained by each PC. (c) PC1 loading plot. (d) PC2 loading plot.

Due to the high dimensionality and complexity of the SERS spectra, it was difficult for researchers to distinguish CRP by relying on visual inspection alone. By using PCA dimensionality reduction, the structure of the spectral data was simplified, retaining major information while reducing the number of features, thereby improving the efficiency and effectiveness of the classification process.^43^, ^44^ PC1 accounted for 85.7% of the total variance, indicating that it captured the primary variations in the spectral data, while PC2 explained 2.4% of the total variance, serving as a complement to the results of PC1 (Figure 5a). Each point represented a sample's SERS spectrum, with most points in each class falling within a distinct 95% confidence ellipse. The plot showed that the distribution of CRC was relatively independent, while the distributions of HC and CRP partially overlapped. The score plot revealed the intrinsic relationships and differences in spectral features among the different sample categories. Figure 5b showed the variance proportion explained by each PC and their cumulative variance explained. It showed that as the number of PCs increased, the cumulative variance explained gradually stabilized, and the first few PCs captured most of the information in the SERS spectra.

Figure 5c shows the PC1 loading plot. PC1 accounted for the largest variance in the spectral data, essential for distinguishing CRP. Key characteristic peaks were observed at positive peaks 747, 835, 1000, 1340, 1450 cm^−1^, and a negative peak at 978 cm^−1^. The 747 and 1340 cm^−1^ peaks reflected changes in nucleic acid structure and content, indicating cancer and CRP‐specific alterations. The 835 and 1000 cm^−1^ peaks showed changes in protein composition and environment, while the 1450 cm^−1^ peak was related to variations in protein content. The 978 cm^−1^ negative peak indicated a reduction in protein and lipid components in CRP patients. These changes were likely caused by metabolic disturbances, abnormal cell proliferation, and differentiation. Figure 5d shows the PC2 loading plot with key characteristic peaks: positive peaks at 673, 978, and 1107 cm^−1^, and negative peaks at 835 and 1650 cm^−1^. The 673 cm^−1^ peak indicated nucleic acid changes, while the 1107 cm^−1^ peak reflected protein structure alterations. The negative peaks at 835 and 1650 cm^−1^ confirmed shifts in protein composition and secondary structures. PC2 served as a supplementary component, further enhancing the identification of CRP. The scatter plots (Figure S1) of key characteristic peaks in PC1 and PC2 loading plots by Student's *t* test (*p < 0.05, **p < 0.01, ***p < 0.005) clearly demonstrated the classification. The intensities in Figure S1 corresponded to the intensities at the key significant characteristic peaks of samples from different stages. These key significant peaks were selected based on the loading scores of PC1 and PC2 and were further confirmed for significance using the Student's *t* test (*p < 0.05, **p < 0.01, ***p < 0.005).

**FIGURE 6:**
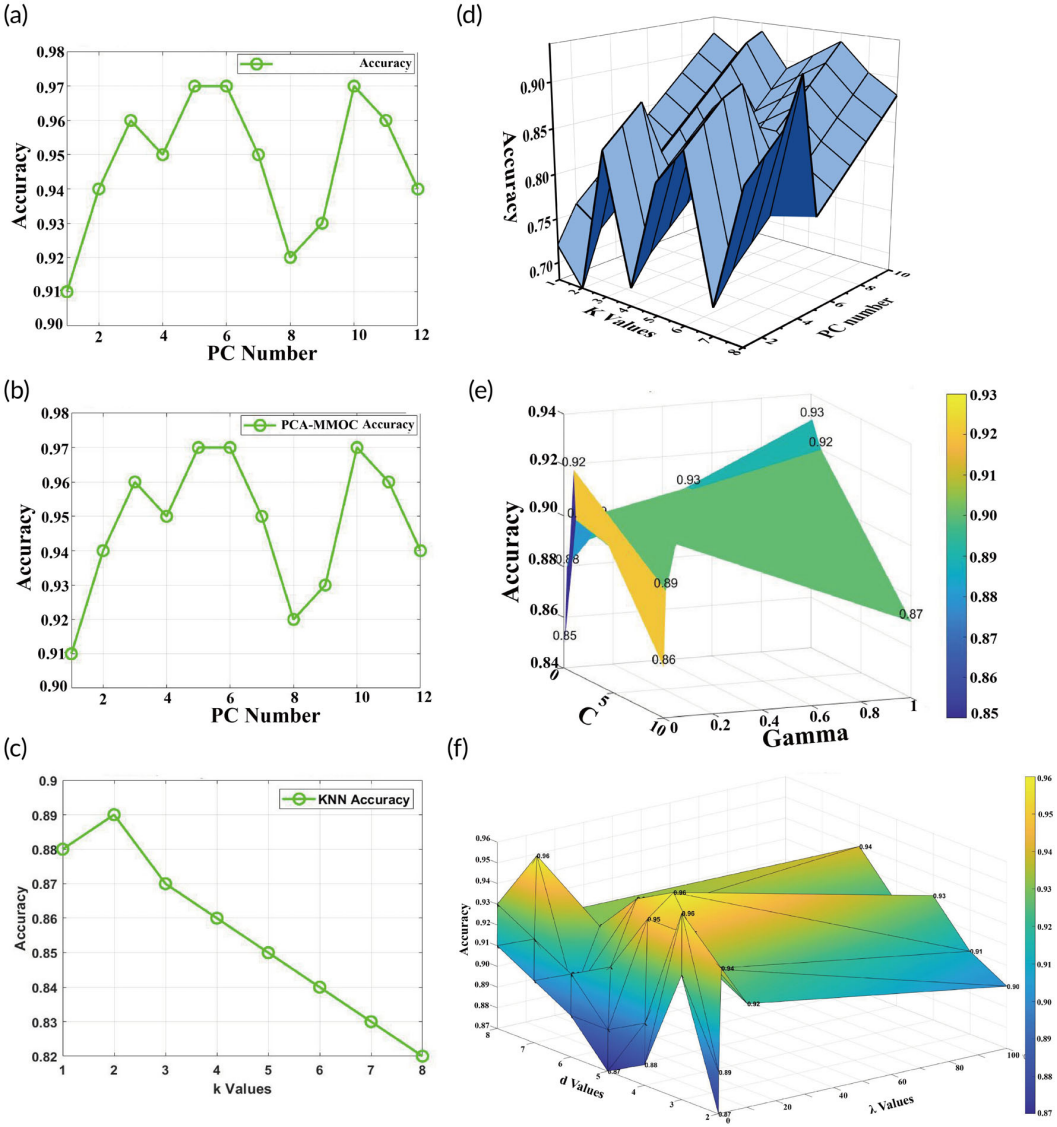
(a) PCA‐OCDCO model accuracy variation under different PCs. (b) PCA‐SVM model accuracy variation under different PCs. (c) KNN model accuracy variation under different K values. (d) PCA‐KNN model accuracy variation under different PCs and K values. (e) SVM model accuracy variation under C and gamma values. (f) OCDCO model accuracy variation under different *d* and *λ* values.

After PCA, the cumulative PCs from 1 to 12 were used as inputs for OCDCO, respectively. OCDCO is an optimization method that enhances classifier accuracy and performance by maximizing inter‐class distance and minimizing intra‐class distance. The principal diagram of the OCDCO algorithm for multi‐class visualization is shown in Figure S2. The diagram displays the optimization process of samples from three different classes in the two‐dimensional space, where intra‐class samples tightly clustered around their respective class means, while inter‐class means gradually separated and maintained maximum distance. Table S2 shows the steps of the OCDCO algorithm. Figure 6a shows that the PCA‐OCDCO model's accuracy fluctuated between 0.91 and 0.97, peaking at 98% with 5 PCs, indicating high classification performance and stability. Figure 6b presents the PCA‐SVM model, with accuracy ranging from 0.91 to 0.96, reaching its best with 4 PCs. Figure 6c depicts the KNN model's accuracy, which decreased from 0.89 to 0.82 as the *K* value increased, showing poor stability. Figure 6d displays the PCA‐KNN model, with accuracy fluctuating between 0.85 and 0.88. Figure 6e illustrates the SVM model's accuracy, ranging from 0.85 to 0.93, dependent on parameter selection. Figure 6f shows the mmoc model's accuracy from 0.86 to 0.91, indicating good stability but lower overall accuracy. In summary, the PCA‐OCDCO and PCA‐SVM models achieved the best classification performance and stability, while KNN and PCA‐KNN showed lower stability.

**FIGURE 7:**
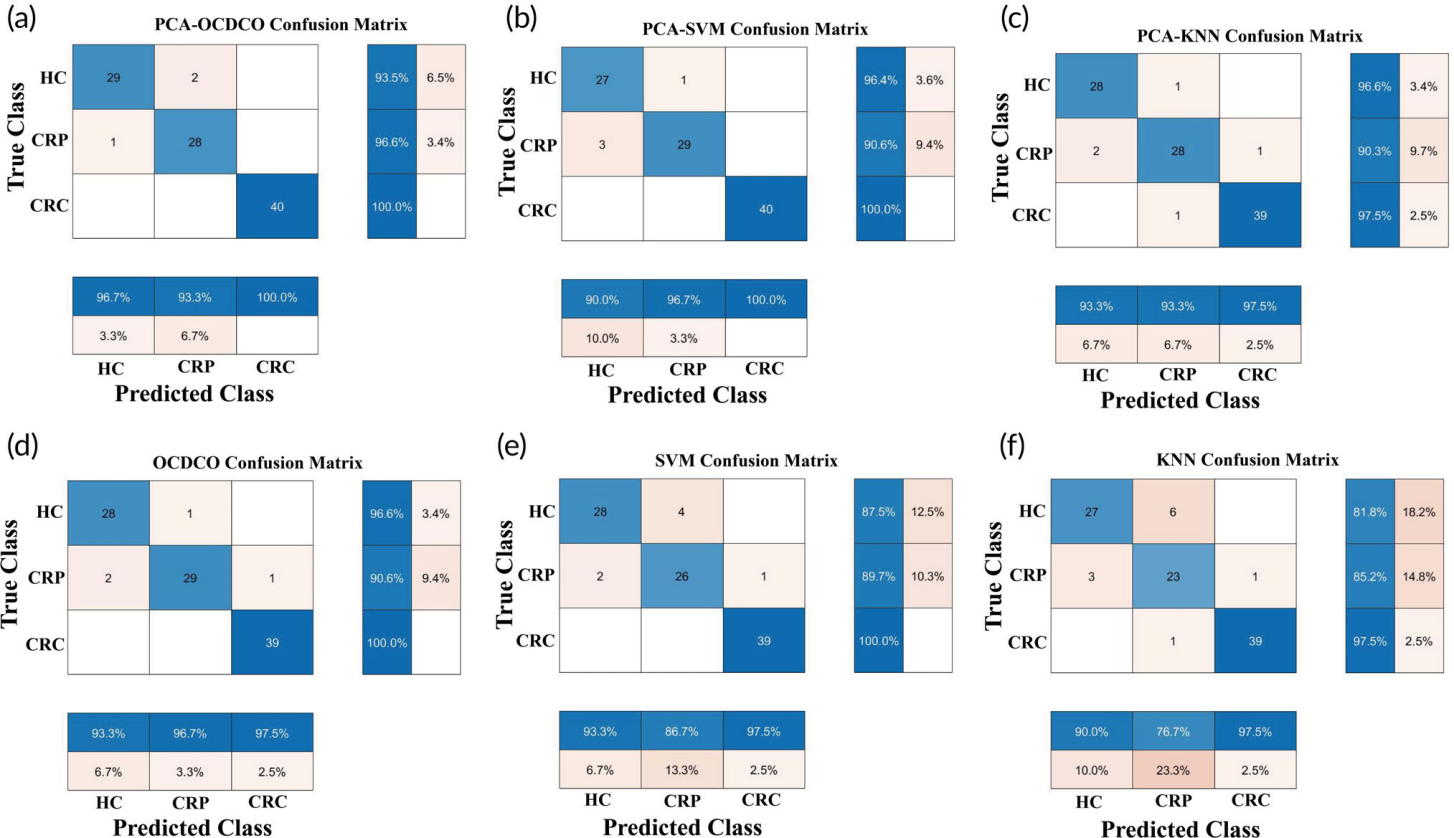
The confusion matrices of (a) PCA‐OCDCO, (b) PCA‐SVM, (c) PCA‐KNN, (d) OCDCO, (e) SVM, and (f) KNN.

Figure 7 displays the confusion matrices for various classification models at their highest accuracy. In the PCA‐OCDCO model (Figure 7a), the HC class had 1 error (96.7% accuracy), CRP had 2 errors (93.3% accuracy), and CRC had no errors (100% accuracy). The PCA‐SVM model (Figure 7b) had 3 errors in HC (90.0% accuracy), 1 in CRP (96.7%), and none in CRC (100%). In the PCA‐KNN model (Figure 7c), HC and CRP had 1 and 2 errors, respectively (96.7%, 93.3%), with 1 error in CRC (97.5%). The OCDCO model (Figure 7d) achieved accuracies of 96.7%, 96.7%, and 97.5%, respectively, across the three classes. In the SVM model (Figure 7e), HC had 4 errors (86.7%), CRP had 2 errors (93.3%), and CRC had 1 error (97.5%). Lastly, the KNN model (Figure 7f) showed the lowest performance, with HC at 80.0%, CRP at 76.7%, and CRC at 97.5%. Overall, PCA‐OCDCO demonstrated the best classification performance, particularly excelling in the CRC class with 100% accuracy.

**FIGURE 8:**
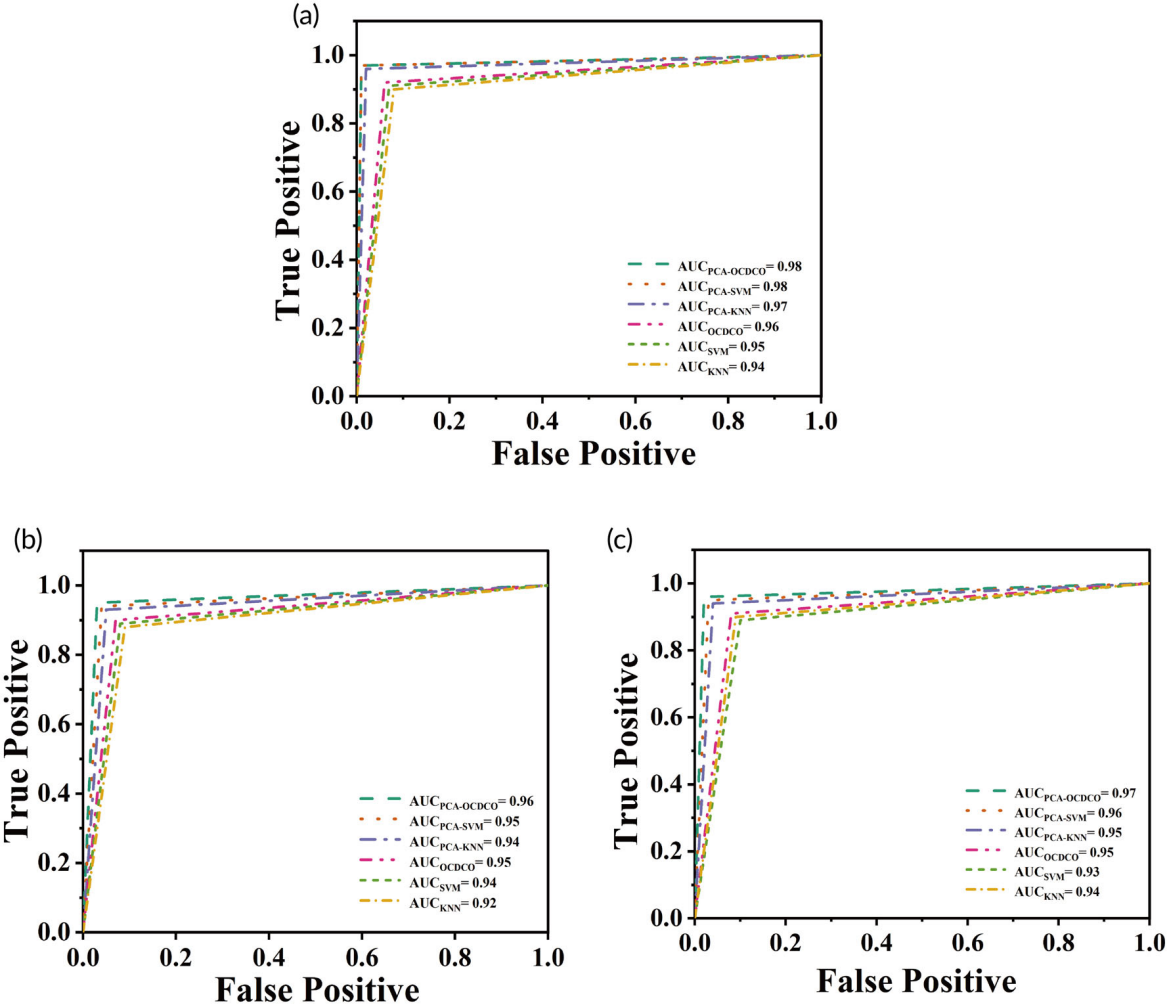
The AUC values of different models for (a) HC versus CRP&CRC, (b) CRP versus HC&CRC, and (c) CRC versus HC&CRP.

Tables S3–S5 shows the sensitivity, specificity, and AUC of different models for HC versus CRP&CRC, CRP versus HC&CRC, CRC versus HC&CRP. Figure 8 displays the AUC values for different models across three data sets (HC vs. (CRP&CRC), CRP vs. (HC&CRC), and CRC vs. (HC&CRP)). In Figure 8a, PCA‐OCDCO and PCA‐SVM achieved the highest AUC of 0.98, followed by PCA‐KNN with 0.97. The AUC for OCDCO, SVM, and KNN was 0.96, 0.95, and 0.94, respectively. Figure 8b shows PCA‐OCDCO in CRP versus (HC&CRC) with an AUC of 0.96, followed by PCA‐SVM and OCDCO at 0.95, while KNN had the lowest at 0.92. Figure 8c shows that the AUC of PCA‐OCDCO was 0.97 in CRC versus (HC&CRP), outperforming others. Overall, PCA‐OCDCO showed the best performance, with PCA‐SVM and PCA‐KNN also performing well.

## 4 CONCLUSIONS

In conclusion, we have developed an innovative approach that integrates SERS technology with the PCA‐OCDCO model for the rapid and label‐free detection of CRP. This method leverages a specially designed microarray chip embedded with an Au/SnO_2_ NRAs substrate, which demonstrates exceptional sensitivity, stability, and uniformity in SERS measurements. Using this chip as the sensing platform, high‐quality SERS spectra were obtained from serum samples of healthy controls, CRP patients, and CRC patients, revealing distinct spectral features associated with nucleic acids, proteins, and lipids. The PCA‐OCDCO model effectively classified these spectra, achieving high accuracy, sensitivity, and specificity. Key spectral features, such as those at 747, 835, 978, 1000, 1340, and 1450 cm^−1^, were identified as critical for distinguishing between different groups. Compared to conventional methods like PCA‐SVM and PCA‐KNN, the PCA‐OCDCO model demonstrated superior performance in handling complex spectral data. This integrated SERS‐PCA‐OCDCO platform represents a robust and efficient tool for label‐free serum analysis in liquid environments, offering significant potential for early disease detection, personalized treatment strategies, and clinical diagnostics. Future studies will focus on validating this approach in larger cohorts and exploring its application in other disease contexts.

## AUTHOR CONTRIBUTIONS

**Qunshan Zhu:** Methodology; validation; formal analysis; investigation; data curation; writing – original draft. **Gaoyang Chen:** Investigation; resources; data curation; writing – original draft. **Lei Fu:** Formal analysis; investigation; data curation; writing – review and editing. **Dawei Cao:** Methodology; software; validation. **Zhenguang Wang:** Methodology; resources; validation. **Yan Yang:** Writing – review and editing; project administration; funding acquisition. **Wei Wei:** Visualization; supervision; project administration; funding acquisition.

## CONFLICT OF INTEREST STATEMENT

The authors declare no competing interests.

## Supporting information


Data S1.

## Data Availability

The data that support the findings of this study are available from the corresponding author upon reasonable request.
