# Supplementary material for: Machine learning‐based SERS serum detection platform for high‐sensitive and high‐throughput diagnosis of colorectal precancerous lesions
Source: Bioeng Transl Med. 2025 Mar 28;10(5):e70019. doi: 10.1002/btm2.70019 (PMC12478330; doi:10.1002/btm2.70019)
Supplement: Supplementary file 1 — Data S1. [file BTM2-10-e70019-s001.docx]

**Supporting Information**

**Machine learning-based SERS serum detection platform for high-sensitive and high-throughput diagnosis of colorectal precancerous lesions**

Qunshan Zhu^1†^**|** Gaoyang Chen^2†^**|** Lei Fu^3^**|** Dawei Cao^4^**|** Zhenguang Wang^1^**|** Yan Yang^1^**|** Wei Wei^1^*

^1^Department of Gastrointestinal Surgery, Jiangdu People's Hospital Affiliated to Yangzhou University, Yangzhou 225200, China

^2^Department of Oncology, The Affiliated Taizhou Second People’s Hospital of Yangzhou University, Taizhou 225300, China

^3^Department of Pathology, Jiangdu People's Hospital Affiliated to Yangzhou University, Yangzhou 225200, China

^4^School of Information Engineering, Yangzhou Polytechnic Institute, Yangzhou 225002, China

*Corresponding authors: jd.k.kenny@163.com

^†^Qunshan Zhu and Gaoyang Chen contributed equally to this paper

**1 | Results and discussion**

**1.1 | SERS spectral analysis of serum samples**

**TABLE S1** The detailed assignments of characteristic peaks

| **Raman shift (cm^-1^)** | **Assignment** | **Reference** |
| --- | --- | --- |
| 673 | Ring breathing modes in the DNA bases G (ring breathing modes in  the DNA bases) | 1 |
| 747 | T (ring breathing mode of DNA/RNA bases) | 1 |
| 835 | Asymmetric O-P-O stretching, tyrosine | 1 |
| 893 | Phosphodiester, Deoxyribose | 2 |
| 978 | C-C stretching β-sheet (proteins), ==CH bending (lipids) | 3 |
| 1000 | Phenylalanine, Bound & free NADH | 4 |
| 1032 | CH_2_CH_3_ bending modes of collagen & phospholipids, Phenylalanine of collagen | 5, 6 |
| 1107 | Phenylalanine (proteins) | 7 |
| 1159 | C-C/C-N stretching (proteins) | 3 |
| 1204 | Tyrosine, phenylalanine | 7 |
| 1253 | C-N stretching | 8 |
| 1340 | Nucleic acid modes indicating the nucleic acid content in tissues | 9 |
| 1450 | CH_2_ bending (proteins) | 7 |
| 1521 | -C==C- Carotenoid | 10 |
| 1611 | Tyrosine | 6 |
| 1650 | (C==C) Amide I, Protein amide I absorption | 4 |
| 1704 | ν(C==O) OH (amino acids aspartic & glutamic acid) | 11 |
| 1741 | Carbonyl feature of lipid spectra | 12 |

**1.2 | Multivariate analysis**





**FIGURE S1** The scatter plots of significant peaks in PC1, and PC2 loading plots for distinguishing SERS spectra of CRP by student t-test (*p < 0.05, **p < 0.01, ***p < 0.005). (A) 673 cm^-1^, (B) 747 cm^-1^, (C) 835 cm^-1^, (D) 978 cm^-1^, (E) 1000 cm^-1^, (F) 1107 cm^-1^, (G) 1340 cm^-1^, (H) 1450 cm^-1^, (I) 1650 cm^-1^.

Figure S2 displayed the principle diagram of the OCDCO algorithm, illustrating the optimization of three classes in 2D space. Intra-class samples clustered around their means, while inter-class means separated to maximize distance. Table S2 showed the steps of the OCDCO algorithm.

1. Input Data

The dataset contains three classes, each with several sample points. These sample points are represented by red, green, and blue dots in the figure.

1. Calculate the Mean Vector for Each Class

Calculate the mean vector for each class:

$\mu_{1}=\frac{1}{\text{ }n_{1}}\sum_{x\in C_{1}} x$, $\mu_{2}=\frac{1}{\text{ }n_{2}}\sum_{x\in C_{2}} x$, $\mu_{3}=\frac{1}{\text{ }n_{3}}\sum_{x\in C_{3}} x$ (1)

Where $n_{i}$ was the number of samples in the i-th class, $C_{i}$ was the sample set of the i-th class, and $\mu_{i}$  was the mean vector of the i-th class.

1. Calculate the Overall Mean Vector

Calculate the overall mean vector of all samples:

$\mu=\frac{1}{\text{ }n}\sum_{i=1}^{n} x_{i}$ (2)

Where *n* was the total number of samples, *μ* was the overall mean vector, represented by the cyan cross in the figure.

1. Calculate the Between-class Scatter Matrix $S_{b}$:

Calculate the between-class scatter matrix based on the scatter between the mean vectors of each class and the overall mean vector:

$S_{b}=\sum_{i=1}^{k} n_{i}(\mu_{i}-\mu)(\mu_{i}-u)^{T}$ (3)

Where $k$ was the number of classes.

1. Calculate the Within-class Scatter Matrix $S_{\text{w}}$:

Calculate the within-class scatter matrix based on the scatter of the samples within each class around their respective mean vectors:

$S_{w}=\sum_{i=1}^{k} \sum_{x\in C_{\text{i}}} (\mu_{i}-\mu)(\mu_{i}-u)^{T}$ (4)

Where $C_{i}$ was the sample set of the i-th class. This matrix measures the scatter of samples within each class around their respective mean vectors.

1. Construct and Optimize the Objective Function:

The objective function aims to maximize the separation between different classes while minimizing the scatter within each class. This is achieved by optimizing a specific ratio that involves the between-class scatter matrix ($S_{b}$) and the within-class scatter matrix ($S_{\text{w}}$):

$\max_{W} \frac{trace (W^{T}S_{b}W)}{trace (W^{T}S_{w}W)}$ (5)

This optimization problem can be solved using eigenvalue decomposition. Specifically, the optimal projection matrix $W$is found by solving the generalized eigenvalue problem

$S_{\text{w}}^{-1}S_{b}W=\text{λ}W$ (6)

Where the *λ* denoted the eigenvalue, and *W* was the corresponding eigenvector. The eigenvectors corresponding to the largest eigenvalues form the columns of the projection matrix *W*.

1. Projection and Classification:

Once the optimal projection matrix $W$ is obtained, each sample $x$ in the dataset is projected into a new space:

$z=W^{T}x$ (7)

Where $z$ was the projected representation of $x$ in the new low-dimensional space. In the new projected space, the samples are classified based on their proximity to the projected mean vectors of each class.

For a new sample$x_{\text{new}}$, the projection is computed as:

$z_{\text{new}}=W^{T}x_{new}$ (8)

Where $z_{new}$​was the projected representation of $x_{new}$ in the low-dimensional space.

**TABLE S2** Steps of the OCDCO Algorithm

| Steps:   1. Input Data   Dataset with multiple classes, each represented by sample points. Each class $C_{i}$ has $n_{i}$ samples, *n* was the total number of samples, and $k$ was the total number of classes.   1. Calculate Mean Vectors   Class mean $\mu_{i}$: $\mu_{\text{i}}=\frac{1}{\text{ }n_{3}}\sum_{x\in C_{i}} x$   Overall mean *μ*: $\mu=\frac{1}{\text{ }n}\sum_{i=1}^{n} x_{i}$    1. Calculate Scatter Matrices   Between-class scatter $S_{b}$: $S_{b}=\sum_{i=1}^{k} n_{i}(\mu_{i}-\mu)(\mu_{i}-u)^{T}$   Within-class scatter : $S_{w}=\sum_{i=1}^{k} \sum_{x\in C_{\text{i}}} (\mu_{i}-\mu)(\mu_{i}-u)^{T}$    1. Optimize Objective Function   Maximize: $\max_{W} \frac{trace (W^{T}S_{b}W)}{trace (W^{T}S_{w}W)}$   Solve: $S_{\text{w}}^{-1}S_{b}W=\text{λ}W$   Select top *k* eigenvectors to form projection matrix *W*.   1. Projection and Classification   Project samples: $z=W^{T}x$   Classify new samples based on the smallest distance to class means in the projected space. |
| --- |


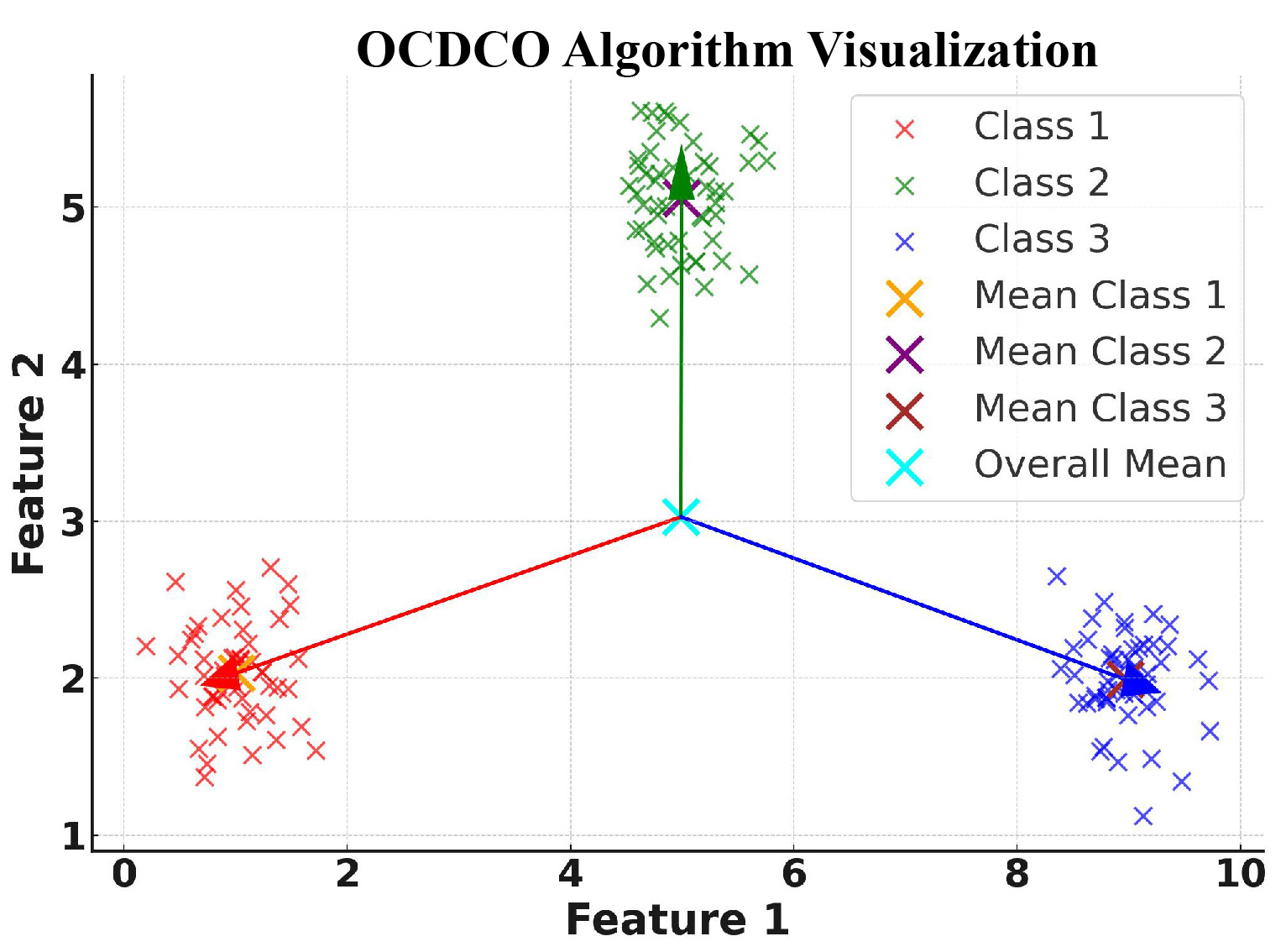


**FIGURE S2** Principle diagram of the OCDCO algorithm for multi-class visualization

**TABLE S3** Sensitivity, Specificity, and AUC of different models for HC vs CRP&CRC

| **Models** | **HC vs (CRP&CRC)** | | | |
| --- | --- | --- | --- | --- |
|  | **Sensitivity** | **Specificity** | **AUC** | **Num_PC** |
| PCA-OCDCO | 97.0% | 99.0% | 0.98 | 10 |
| PCA-SVM | 97.0% | 99.0% | 0.98 | 15 |
| PCA-KNN | 96.0% | 98.0% | 0.97 | 12 |
| OCDCO | 92.0% | 94.0% | 0.96 | N/A |
| SVM | 91.0% | 93.0% | 0.95 | N/A |
| KNN | 90.0% | 92.0% | 0.94 | N/A |
| PLS-DA | 94.0% | 97.0% | 0.96 | N/A |

**TABLE S4** Sensitivity, Specificity, and AUC of different models for CRP vs HC&CRC

| **Models** | **CRP vs HC&CRC** | | | |
| --- | --- | --- | --- | --- |
|  | **Sensitivity** | **Specificity** | **AUC** | **Num_PC** |
| PCA-OCDCO | 95.0% | 97.0% | 0.96 | 7 |
| PCA-SVM | 94.0% | 96.0% | 0.95 | 10 |
| PCA-KNN | 93.0% | 95.0% | 0.94 | 11 |
| OCDCO | 90.0% | 93.0% | 0.95 | N/A |
| SVM | 89.0% | 92.0% | 0.94 | N/A |
| KNN | 88.0% | 91.0% | 0.92 | N/A |
| PLS-DA | 93.0% | 94.0% | 0.94 | N/A |

**TABLE S5** Sensitivity, Specificity, and AUC of different models for CRC vs HC&CRP

| **Models** | **CRC vs (HC&CRP)** | | | | |
| --- | --- | --- | --- | --- | --- |
|  | **Sensitivity** | **Specificity** | **AUC** | **Num_PC** |  |
| PCA-OCDCO | 96.0% | 98.0% | 0.97 | 9 |  |
| PCA-SVM | 95.0% | 97.0% | 0.96 | 13 |  |
| PCA-KNN | 94.0% | 96.0% | 0.95 | 6 |  |
| OCDCO | 91.0% | 92.0% | 0.95 | N/A |  |
| SVM | 89.0% | 90.0% | 0.93 | N/A |  |
| KNN | 90.0% | 91.0% | 0.94 | N/A |  |
| PLS-DA | 94.0% | 96.0% | 0.95 | N/A |  |

**REFERENCES**

1. Chan JW, Taylor DS, Zwerdling T, et al. Raman Spectroscopy for Cellular Analysis. *Biophys J.* 2006; 90 (2): 648-656.
2. Ruiz-Chica AJ, Medina MA, Sanchez-Jimenez F, et al. Raman Spectroscopy Study of Cell Cultures. *J Raman Spectrosc.* 2004; 35 (1): 93-100.
3. Notingher I, Green C, Dyer C. Non-invasive Spectroscopy for Cellular Analysis. *J R Soc Interface.* 2004; 1 (2): 79-90.
4. Malini R, Venkatakrishma K, Kurien J, et al. Raman Spectroscopic Study of Biopolymers. *Biopolymers.* 2006; 81 (3): 179-193.
5. Huang Z, McWilliams A, Lui M, et al. Raman Spectroscopy of Human Tissues. *Int J Cancer.* 2003; 107 (5): 1047-1052.
6. Cheng WT, Liu MT, Liu HN, et al. Applications of Raman Spectroscopy in Microscopy. *Microsc Res Tech.* 2005; 68 (1): 75-79.
7. Lakshimi RJ, Kartha VB, Krishna CM, et al. Spectroscopic Analysis of Tissues. *Radiat Res.* 2002; 157 (2): 175-182.
8. Naumann D. Fourier Transform Infrared Spectroscopy of Biological Cells. *Proc SPIE.* 1998; 3257 (4): 245-257.
9. Stone N, Kendall C, Smith J, et al. Raman Spectroscopy for Cancer Diagnosis. *Faraday Discuss.* 2004; 126 (1): 141-157.
10. Fung MFK, Senterman MK, Mikhael NZ, et al. Raman Spectroscopic Analysis of Cervical Cancer. *Biospectroscopy.* 1996; 2 (2): 155-165.
11. Shetty G, Kendall C, Shepherd N, et al. Applications of Raman Spectroscopy in Gastrointestinal Cancer. *Br J Cancer.* 2006; 94 (10): 1460-1464.
12. Kline NJ, Treado PJ. Raman Spectroscopy of Biological Tissues. *J Raman Spectrosc.* 1997; 28 (2-3): 119-124.
